# Gynæcological Wards

**Published:** 1871-10

**Authors:** 


### GYNÆCOLOGICAL WARDS.

---

### Service of PROF. BYFORD.

---

CASE I. PERIMETRITIS.—This lady presents a condition not unfrequent, though its cause is perplexing. She married thirteen years ago, at the age of 16. Her health was perfect until the birth of her first child, but ever since she has suffered from dysmenorrhœa and leucorrhœa between the molimena. She says that four years ago she was treated for inflammation of the bowels, and at the time of the fire in this city she was again attacked by the same symptoms as before, and having no home left, she entered the hospital. She had fever, pain in the back, hips and abdomen, pain in urinating, nausea and and vomiting. The fever was considered to be an intermittent by a physician who saw her before she came to the Hospital. The house physician here prescribed paregoric and nitre, on account of the pain in the bladder, believing she had cystitis, and for the nausea and vomiting he gave her carbolic acid dissolved in glycerine and water. These medicines effectually relieved the symptoms before the patient was seen by the Professor. On examining her, he at once recognized a case of perimetritus. A lump hard as bone, and very tender under pressure, was felt in the left iliac region, extending diagonally into the other iliac region. By a vaginal examination a hard tumor was found filling the pelvis and pressing against the bladder. The consequent inflammation explains the pain in urinating; but besides this tumefaction of the uterus, the pelvic cavity was also filled by a fibrinous exudation.

A mistake in diagnosis is liable to be made by almost any one, especially since examination is so frequently neglected. The chills, fever and perspiration occurring every morning, made up apparently a good case of intermittent fever for the physician who first saw her; but the dysury and pains ought to have induced an examination. External exploration should be made in search of hard, resisting masses. Hard tumors can be distinguished by their giving dullness on percussion, whereas the intestines give resonance on percussion. Of course a vaginal examination should be made. The extreme frequency of these cases is often overlooked, because examination is neglected.

The patient was much prostrated, but was considerably relieved by the medicines already noticed. Fomentations of aconite and arnica leaves were directed, and likewise poultices of corn meal or flax-seed. The mixture of muriate of ammonia, tartarized antimony and morphia, was also ordered to be given four times a day. She is improving rapidly, and is about ready to go home. At present, 2½ weeks after her entrance into the Hospital, examination shows that the pelvic mass is gone, and there is no tumefaction, except in the uterus, which is moveable.

